# Variation in survival after surgery for peri-ampullary cancer in a regional cancer network

**DOI:** 10.1186/s12893-017-0220-3

**Published:** 2017-03-07

**Authors:** Bassem Amr, Golnaz Shahtahmassebi, Somaiah Aroori, Matthew J. Bowles, Christopher D. Briggs, David A. Stell

**Affiliations:** 1 Peninsula HPB Unit, Level 7, Derriford Hospital, Derriford Road, Plymouth, Devon PL6 8DH UK; 2 Peninsula Schools of Medicine and Dentistry, Plymouth University, Plymouth, Devon PL6 8BU UK; 3 School of Science and Technology, Nottingham Trent University, Nottingham, NG1 4BU UK

**Keywords:** Ampulla, Bile duct, Pancreatic, Cancer, Centralized hospital services

## Abstract

**Background:**

Centralisation of specialist surgical services requires that patients are referred to a regional centre for surgery. This process may disadvantage patients who live far from the regional centre or are referred from other hospitals by making referral less likely and by delaying treatment, thereby allowing tumour progression. The aim of this study is to explore the outcome of surgery for peri-ampullary cancer (PC) with respect to referring hospital and travel distance for treatment within a network served by five hospitals.

**Methods:**

Review of a unit database was undertaken of patients undergoing surgery for PC between January 2006 and May 2014.

**Results:**

394 patients were studied. Although both the median travel distance for patients from the five hospitals (10.8, 86, 78.8, 54.7 and 89.2 km) (*p* < 0.05), and the annual operation rate for PC (2.99, 3.29, 2.13, 3.32 and 3.07 per 100,000) (*p* = 0.044) were significantly different, no correlation was noted between patient travel distance and population operation rate at each hospital. No difference was noted between patients from each hospital in terms of resection completion rate or pathological stage of the resected tumours. The median survival after diagnosis for patients referred from different hospitals ranged from 1.2 to 1.7 years and regression analysis revealed that increased travel distance to the regional centre was associated with a small survival advantage.

**Conclusion:**

Although variation in the provision and outcome of surgery for PC between regional hospitals is noted, this is not adversely affected by geographical isolation from the regional centre.

**Trial registration:**

This study is part of post-graduate research degree project. The study is registered with ClinicalTrials.gov (unique identifier NCT02296736) November 18, 2014.

## Background

Since publication of the Improving Outcomes Document in September 2000 [1] surgery for periampullary cancer (PC) in the UK has been centralised into designated regional Hepato-Pancreatico-Biliary (HPB) centres, each serving a population of approximately two million. This process requires that most hospitals do not undertake pancreatic resection, but perform the initial treatment and assessment of patients with potential PC, before referral to the regional tertiary centre. This separation of secondary from tertiary care in different hospitals has the potential to disadvantage patients referred from hospitals other than the regional centre, as the referral process is likely to be more complex than when secondary and tertiary care are provided on the same site. Inevitably provision of pancreatic surgical services in a single HPB centre within a large area will impose greater difficulty and inconvenience for some patients in travelling to the regional centre, which may adversely affect referral for treatment for patients with PC. Furthermore delays in treatment for patients residing further from the regional centre may allow tumour progression and have an adverse effect on outcomes.

The potential influence of referral between hospitals and geographical isolation on the outcome of surgery for PC has not been assessed and the aim of this study is to assess associations between referring hospital of origin and traveling distance to the regional HPB surgical centre with the population rate of surgery for PC, the interval to surgery, pathological outcome and long-term survival after diagnosis of PC within a cancer network.

## Methods

The Peninsula HPB unit provides pancreatic surgical services to the Peninsula Cancer Network, which serves the largely rural UK counties of Devon and Cornwall, ranking the 7^th^ and 12^th^ least densely populated of 90 English local government areas [2]. The population of the two counties (1.67 million) is served by four hospitals providing secondary care only, and one hospital which provides secondary care and also hosts the regional tertiary HPB surgery centre. Surgery and immediate post-operative care are provided by the regional centre. All other treatment including stent insertion, adjuvant chemotherapy and long-term follow-up are provided by local hospitals. All hospitals are linked by a weekly audio-visual MDT with the regional centre. Referral and transfer of patients follows agreed protocols and is coordinated by nurse specialists.

Details of a consecutive series of patients having surgery at the Peninsula HPB unit between January 2006 and May 2014 were studied. Demographic, operative and pathology data were retrieved from the unit database. Included patients were those who underwent surgery for PC where final histology revealed a diagnosis of pancreatic, ampullary, distal bile duct or duodenal adenocarcinoma, or those where resection could not be completed and intra-operative biopsy confirmed the presence of adenocarcinoma. Patients receiving neo-adjuvant chemotherapy were excluded. The size of the catchment area served by each of the hospitals in the Peninsula was obtained from South West Public Health Observatory [3]. The travel distance by road for each patient was obtained from the AA mileage calculator (with permission) using post-code data [4]. The interval to surgery was calculated from the date of diagnosis of PC, which was taken as the date of the first cross-sectional abdominal imaging which suggested this diagnosis. The presence of biliary obstruction was defined as either clinically evident jaundice at the time of surgery or the requirement for pre-operative biliary drainage. Pre-operative diabetes was defined as the requirement for hypoglycaemic medication. The workload in the HPB surgical centre is shared non-selectively by four surgeons and is undertaken using standardised techniques, and in-patient care follows a standard protocol. The American Society of Anaesthesiologists (ASA) grade was determined at the time of surgery by the responsible anaesthetist. Resected specimens were analysed according to Royal College of Pathologists guidelines [5] and the TNM classification systems [6] was used to describe pathological stage. Survival data were obtained from hospital and general practice records and included all deaths occurring after surgery, including in-hospital mortality. Survival times were calculated to include the interval prior to surgery and therefore were taken from the date of the first cross-sectional image which raised the suspicion of PC. Survival data for the whole group of patients referred from each hospital is given as single outcome of interest and is reported as median and range. Follow-up was completed 1^st^ May 2015.

Differences in demographics, operation rates, travel distance, interval to surgery and pathology outcome were compared between hospitals (pathology results for patients with duodenal cancer were not included due to low numbers). Difference in discrete variables was assessed by Pearson Chi square test and continuous variables by Kruskal-Wallis test. Correlation was assessed by Spearman correlation coefficient. To explore potential associations with patient survival a Cox regression analysis of pre-operative factors including age, gender, ASA grade, travel distance and the presence of biliary obstruction at presentation was undertaken. In addition, patient survival across five hospitals was compared using Kaplan–Meier survival curves and between hospital pairs by Cox regression analysis.

## Results

**Fig. 1:**
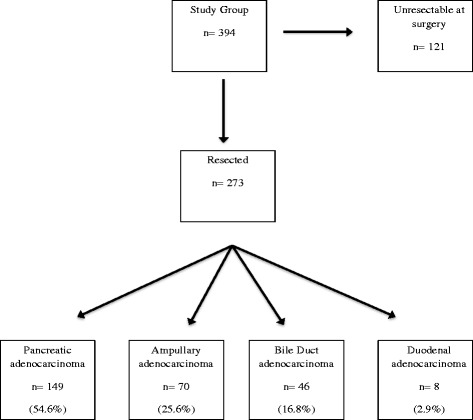
Patients undergoing surgery for PC at Peninsula HPB Centre between January 2006 and May 2014

During the study period 394 patients fulfilling the study criteria underwent surgery to attempt resection of PC at the regional HPB surgery centre (hospital A) (Fig. 1). The median age (66.7 years, range 39.4- 86.4) and gender mix (56.3% male) of the whole group did not vary between patients referred from hospital A, or from hospitals providing secondary care only (hospital B to E) (Table 1). The number of operations for PC undertaken as a proportion of the local population however varied significantly between referring hospitals (Table 1). The median distance patients were required to travel for care was 61.4 km and was significantly less for patients referred from within the catchment area of the regional HPB surgery centre to that for patients referred from all other hospitals in the Peninsula. No correlation was noted between the median travel distance to the regional centre of patients from the referring hospitals and the operation rate at that hospital (*p* = .855). The second lowest population operation rate was noted from the population receiving secondary care from the hospital hosting the regional HPB centre.

**Table 1:** Details of 394 patients undergoing surgery for peri-ampullary cancer between January 2006 and May 2014, displayed by referring hospital of origin. Hospital A hosts the regional HPB cancer centre

Referring hospital		(A)	(B)	(C)	(D)	(E)	*P*
*n* = 394 (%)		111 (28.2)	97 (24.6)	70 (17.8)	74 (18.8)	42 (10.6)	
Population served		464,437	368,313	410,213	278,555	171,227	
Annual operation rate for PC per 100000		2.99	3.29	2.13	3.32	3.07	0.044
Median Travel Distance (kilometres) (range)		10.8 (2.4–112)	85.9 (45.2–155.8)	78.8 (10.1–130.3)	54.7 (2.4–96.2)	98.3 (63–138.6)	.000
Median age (range)		65.7 (41.2–82.0)	68.4 (41.7–84.0)	65.5 (39.4–78.6)	65.6 (45.9–86.4)	70.2 (50.7–84.4)	.105
Gender (% Male)		53.2	58.8	58.6	58.1	52.4	.880
ASA Grade (%)	1	8 (7.2)	8 (8.2)	8 (11.4)	7 (9.5)	0	.416
	2	56 (50.5)	53 (54.6)	39 (55.7)	41 (55.4)	22 (52.4)	
	3	28 (25.2)	26 (26.8)	18 (25.7)	18 (24.3)	14 (33.3)	
	4	2 (1.8)	1 (1)	0	0	0	
	Missing	17 (15.3)	9 (9.3)	5 (7.1)	8 (10.8)	6 (14.3)	
Diabetes	Yes (%)	13 (11.7)	10 (10.3)	7 (10.0)	6 (8.1)	5 (11.9)	.987
	Missing data	12 (10.8)	17 (17.5)	14 (20.0)	15 (20.3)	4 (9.5)	
Jaundice at Presentation (%)		91 (82.0)	82 (84.5)	56 (80)	65 (87.8)	36 (85.7)	.641
Median interval to surgery (days) (range)		47 (5–551)	52 (1–459)	56.5 (16–379)	47 (16–246)	51.5 (6–477)	.108
Resection completed (%)		73 (65.7)	68 (70)	51 (72.8)	51 (68.9)	30 (71.4)	.880
30-day mortality (%)		4 (3.6)	1 (1)	2 (2.8)	1 (1.3)	2 (4.7)	.610

The distribution of ASA grades, the proportion of patients with diabetes, biliary obstruction at the time of surgery and pre-operative biliary intervention did not differ between hospitals (Table 1). The median interval from first investigation suggesting a diagnosis of PC to surgery was 49 days (interquartile range 34–69 days) and was similar between referring hospitals. Correlation analysis revealed no association between the travel distance to the regional HPB surgery centre and the interval to surgery (*p* = .15). In-patient 30-day mortality occurred in 10 (2.5%) patients and did not differ between hospitals.

Tumour resection was completed in 273 patients (69.3%) and the completion rate did not differ between hospitals (Table 2). In 121 patients the tumour was inoperable at the time of surgery either due to the presence of vascular invasion (70) or distant metastases (47). In four patients the reason for irresectability was not recorded. Histological diagnoses of the resected specimens are shown in Fig. 1. Analysis of pathological outcomes revealed no difference between patients from the referral zone of the regional centre and those from other hospitals in the region, in terms of resection completion rate, tumour size, nodal status and resection margin status (Table 2). Similarly the distribution of the main diagnoses of PC did not differ between patients from the regional centre and those from other hospitals.

**Table 2:** Histopathological stage for 265 patients undergoing resection of pancreatic, ampullary and distal bile duct cancer at the regional HPB centre (A) displayed by referring hospital of origin

*N* = 265	A 111	B 97	C 70	D 74	E 42	*P*
Pancreatic cancer (*n* = 149)	40	38	22	28	21	
T size (mm) (range)	30 (15–48)	31.50 (16–60)	30.5 (15–70)	32.5 (12–50)	30 (18–65)	.620
N1disease (%)	35 (87.5)	33 (86.8)	19 (86.4)	23 (82.1)	17 (81)	.940
R1 resection (%)	34 (85)	24 (63.1)	18 (81.8)	24 (85.7)	19 (90.5)	.052
Ampullary cancer (*n* = 70)	21	18	12	13	6	
T size (mm) (range)	25 (12–80)	22.5 (5–65)	23.5 (15–60)	22 (11–65)	28 (8–50)	.933
N1disease (%)	14 (66.6)	10 (55.5)	6 (50)	5 (38.5)	4 (66.6)	.551
R1 resection (%)	7 (33.3)	1 (5.5)	2 (16.6)	2 (15.4)	2 (33.3)	.230
Bile duct cancer (*n* = 46)	10	10	13	10	3	
T size (mm) (range)	25.5 (10–70)	27 (10–45)	25 (10–40)	20 (12–50)	15 (12–20)	.216
N1disease (%)	7 (70)	7 (70)	4 (30.7)	7 (70)	1 (33.3)	.172
R1 resection (%)	5 (50)	6 (60)	5 (38.5)	5 (50)	2 (66.6)	.839

After a median follow-up of 4.5 years (1.3–9.5 years) the median survival (range) of the study group was 1.45 (0.11 – 9.4) years and was similar in males (1.44, 0.13–9.3 years) and females (1.45, 0.11–8.7 years). Two patients were lost to follow-up. Survival was greater in patients where resection was completed (1.85, 0.14–9.4 years) than in those where the tumour could not be removed (0.9, 0.11–2.8 years). The median survival of patients travelling more than the median distance for treatment was 1.5 (0.14–8.7) years compared to 1.4 (0.11–9.4) years for those travelling less than the median travel distance (*p* = 0.234). Cox regression analysis of the association of pre-operative variables including individual patient travel distance however revealed a significant survival advantage associated with increased travel distance to the regional HPB centre (Table 3).

**Table 3:** Cox regression analysis of potential association of pre-operative factors including travel distance to regional HPB centre with survival after diagnosis for 394 patients undergoing surgery for periampullary cancer

		Hazard Ratio	Lower .95	Upper .95	*P*-value
Gender		0.956	0.744	1.229	0.728
Age		1.009	0.995	1.022	0.217
Distance (km)		0.996	0.993	0.999	0.029
Jaundice		0.967	0.686	1.364	0.852
ASA	1 vs 2	0.945	0.678	1.317	0.739
	2 vs 3 & 4	1.117	0.888	1.407	0.344

**Fig. 2:**
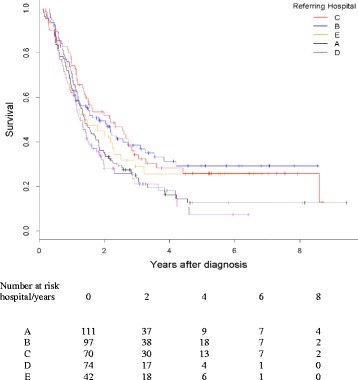
Survival from diagnosis of 394 patients undergoing surgery for periampullary cancer at Peninsula HPB surgery centre between January 2006 and May 2014, according to hospital of referral (*p* = 0.032)

Further survival analysis revealed that the referring hospital of origin was associated with outcome (Fig. 2), with median survival ranging from 1.2 (0.14–6.4) years (patients from hospital D) to 1.5 (0.3–8.8) years (patients from hospital B). Pair by pair regression analysis comparing patients from the catchment area of the regional HPB centre revealed no difference in survival from diagnosis for patients from three hospitals C, D and E, but confirmed the significantly decreased hazard ratio of death of patients referred from hospital B (Table 4).

**Table 4:** Paired regression analysis of association of hospital of referral (B to E) with survival compared to referral from Hospital A among 394 patients undergoing surgery for peri-ampullary cancer

A vs	Hazard Ratio	Lower .95	Upper .95	*P*-value
B	0.6934	0.5011	0.9594	**0.0271**
C	0.7042	0.4952	1.0013	0.0508
D	1.1121	0.7983	1.5493	0.5299
E	0.8228	0.5435	1.2456	0.3565

The data bolded shows a significant findings

## Discussion

The main findings of this study are: 1) within the Peninsula Cancer Network the population operation rate for PC varies significantly between hospital catchment areas but this variation is not related to travel distance to the regional HPB surgical centre and 2) individual patient travel distance to the regional centre does not adversely affect the time to surgery, pathological outcome or survival in patients with PC and 3) the provision of secondary and tertiary care in different hospitals does not adversely affect patient outcomes.

Centralisation of pancreatic surgical services has led to improved outcomes including higher resection rates [7, 8], lower operative mortality [9, 10] and improved long-term survival [11]. Similar improvements with centralisation have been noted for liver [12], oesophageal [13], complex urological [14] and vascular surgery [15]. Despite these findings the population benefits of regionalisation are more difficult to demonstrate. Although studies using hospital data have demonstrated improved outcomes associated with centralisation of surgical services for patients who receive treatment [8, 16, 17], these studies may be biased by selection of patients at the regional centres and do not take into account patients who are not referred for treatment. Studies demonstrating improved population outcomes as a result of regionalisation of complex surgery are more difficult to undertake. The potential disadvantages of centralisation of services include a more complex referral pathway when secondary and tertiary care are provided in different hospitals, and an increased burden of travel for patients living further from the centre, which may discourage referral and attendance for treatment. These consequences of centralisation have been noted [18, 19] and the potential risk is greatest in areas of dispersed population. This has led to controversy over the implementation of centralisation of surgical services in rural communities [20], where the risk of limitation of access due to distance may outweigh the benefit of improved technical outcomes. The observation that operation rates are not adversely affected by distance to the HPB surgical centre, or by referral from a different hospital, and that travel distance itself does not influence the outcome of surgery for PC are important, as they show that regionalisation of surgical services does not necessarily lead to limitations in access or increased patient selection at the HPB surgical centre.

The small variation in operation rate noted between hospitals may reflect differences in levels of comorbidity and suitability for surgery, but may be due to different referral practices within each hospital. The observation that the referring hospital of origin is also associated with long-term survival after surgery for PC is therefore an interesting new finding. Many factors contribute to variation in local survival rates and levels of comorbidity are likely to play a major role. It is interesting to note however that long-term survival is lowest in patients from the hospital with the highest population rate of surgery for PC. This may result from referral of more marginal cases, which is not revealed by the measures of comorbidity and tumour burden used in this study. Variation in population operation rate for PC may also explain some of the variation noted in outcome between high-volume hospitals undertaking pancreatic surgery [21].

The strength of this analysis lies in the accurate collection of individual travel distance to the regional HPB surgery centre in a large consecutive series, and its correlation with prospectively audited outcomes. In this study a single measure of survival of all patients has been used, without division by diagnosis, to allow simple comparison between hospitals. This figure includes deaths due to surgical complications, which accounts for the short survival in some patients. A weakness of the study lies in the characterisation of comorbidity. A more discriminating scoring system is required to investigate the potential association of comorbidity with variations in population operation rate for PC. The relatively long median interval to surgery noted in this study, even for patients with biliary obstruction (47 days), is accounted for by the increasing complexity in the patient pre-operative pathway. This pathway however imposes a similar interval to surgery on patients regardless of geographical isolation from the regional centre. In a small number of patients a long interval to surgery was due to investigations being undertaken in patients with self-resolving jaundice, which was not pursued due to patient improvement.

## Conclusion

This study confirms that centralisation of HPB surgical services can be implemented without imposing disadvantage in surgical outcomes on patients due to travel distance to the HPB surgical centre or referral between hospitals for treatment.
